# Imaging of Fibroblast Activation Protein Alpha Expression in a Preclinical Mouse Model of Glioma Using Positron Emission Tomography

**DOI:** 10.3390/molecules25163672

**Published:** 2020-08-12

**Authors:** Darpan N. Pandya, Akesh Sinha, Hong Yuan, Lysette Mutkus, Kristina Stumpf, Frank C. Marini, Thaddeus J. Wadas

**Affiliations:** 1 Department of Radiology, University of Iowa, Iowa City, IA 52242, USA; darpan-pandya@uiowa.edu (D.N.P.); akesh-sinha@uiowa.edu (A.S.); 2 Department of Radiology, University of North Carolina at Chapel Hill, Chapel Hill, NC 27599, USA; yuanh@med.unc.edu; 3 Department of Regenerative Medicine, Wake Forest University Health Sciences, Winston-Salem, NC 27157, USA; lmutkus@wakehealth.edu (L.M.); kstumpf@wakehealth.edu (K.S.); fmarini@wakehealth.edu (F.C.M.)

**Keywords:** Zirconium-89, fibroblast activation protein alpha, PET, cancer, glioma

## Abstract

Glioblastoma multiforme (GBM) is the most aggressive glioma of the primary central nervous system. Due to the lack of effective treatment options, the prognosis for patients remains bleak. Fibroblast activation protein alpha (FAP), a 170 kDa type II transmembrane serine protease was observed to be expressed on glioma cells and within the glioma tumor microenvironment. To understand the utility of targeting FAP in this tumor type, the immuno-PET radiopharmaceutical [^89^Zr]Zr-Df-Bz-F19 mAb was prepared and Lindmo analysis was used for its in vitro evaluation using the U87MG cell line, which expresses FAP endogenously. Lindmo analysis revealed an association constant (K_a_) of 10^−8^ M^−1^ and an immunoreactivity of 52%. Biodistribution studies in U87MG tumor-bearing mice revealed increasing radiotracer retention in tumors over time, leading to average tumor-to-muscle ratios of 3.1, 7.3, 7.2, and 8.3 at 2, 24, 48 and 72 h, respectively. Small animal PET corroborated the biodistribution studies; tumor-to-muscle ratios at 2, 24, 48, and 72 h were 2.0, 5.0, 6.1 and 7.8, respectively. Autoradiography demonstrated accumulated activity throughout the interior of FAP^+^ tumors, while sequential tumor sections stained positively for FAP expression. Conversely, FAP^−^ tissues retained minimal radioactivity and were negative for FAP expression by immunohistochemistry. These results demonstrate FAP as a promising biomarker that may be exploited to diagnose and potentially treat GBM and other neuroepithelial cancers.

## 1. Introduction

Gliomas, which are brain tumors thought to originate from neurological progenitor cells, represent a diverse group of central nervous system cancers including astrocytomas, oligodendromas, ependymomas and the most aggressive, glioblastoma multiforme (GBM) [1,2,3]. Despite the use of surgery, radiotherapy and pharmacotherapy, prognosis for patients remains poor. This has led researchers to identify and validate new biomarkers that may be exploited for imaging and therapy so that patient outcomes will improve.

The dipeptidyl peptidase (DPP) family of proteins, which includes DPP4, DPP8, DPP9, and fibroblast activation protein alpha (FAP), catalyze the hydrolysis of penultimate prolyl bonds at the N-terminus of proteins [4]. However, FAP, which is a 170 kDa type II transmembrane serine protease, is unique among this enzyme family because of its endopeptidase activity and substrate selectivity. Moreover, unlike other members of this protein family, FAP expression was observed to be negligible in normal adult tissues, but is prominently expressed on the cell surface of neuroepithelial cancer cells, on tumor associated fibroblasts in over 90% of epithelial cancers and several other pathologies [4,5,6,7,8,9]. Accordingly, several reports that describe strategies to target FAP expression for imaging and therapy using peptides [10,11,12], antibodies [13,14,15,16,17,18,19,20], antibody fragments [6,21,22,23], nanoparticles [24] and small molecules [25,26,27,28,29,30,31,32,33,34] have appeared in the literature.

With respect to neuroepithelial cancers, Mentlein and colleagues, using quantitative reverse transcriptase PCR (RT-PCR) and immunohistochemistry, determined that FAP expression was elevated in several glioma subtypes [5,35]. Moreover, data revealed that FAP enabled glioma cell invasion through brain tissue suggesting its role in tumor cell invasion by facilitating the degradation of the brain parenchyma. Additionally, while examining human tumor samples and tumor cells lines to understand the relevance of FAP expression, Busek and colleagues discovered elevated levels of FAP protein in most human high-grade gliomas with a mesenchymal subtype and in several glioma tumor cells lines [36,37,38,39]. Furthermore, their studies indicated that FAP expression correlated with the activation of genes associated with extracellular matrix remodeling and inflammation suggesting that there may be a link between FAP expression, and the aggressive tissue remodeling, necrosis and inflammatory infiltrates observed in glioma tumors exhibiting a mesenchymal phenotype. Finally, they also found FAP expression on a variety of stromal cell populations within these tumors suggesting that targeting FAP for imaging and therapy may provide a comprehensive treatment strategy that simultaneously targets tumor cells and the pro-tumorigenic microenvironment of these neuroepithelial cancers.

Over the last three decades, ^89^Zr has increasingly been used to develop PET radiopharmaceuticals because (1) it has favorable decay characteristics for PET imaging, (2) it is routinely produced at private and academic institutions, (3) it has a half-life compatible with that of circulating antibodies (mAbs), and (4) the bifunctional chelator p-isothiocyanatobenzyl-desferrioxamine B (Df-Bz-NCS), which conjugates with mAbs and chelates ^89^Zr, is readily available to the research community [40,41]. In this report, we describe the synthesis of the PET radiopharmaceutical, [^89^Zr]Zr-Df-Bz-F19 mAb, which is the radiolabeled version of the anti-FAP monoclonal antibody F19 [16,17,19,20]. Additionally, we evaluate its ability to bind the FAP antigen in vitro using Lindmo analysis and in vivo using small animal PET imaging studies. Furthermore, we demonstrate that the Cerenkov luminescence imaging (CLI) of FAP expression using the Cerenkov radiation, which is emitted by this radiopharmaceutical, and standard optical imaging techniques is possible [42,43,44].

## 2. Results

### 2.1. [^89^Zr]Zr-Df-Bz-F19 Preparation and In Vitro Characterization

**Scheme 1:**
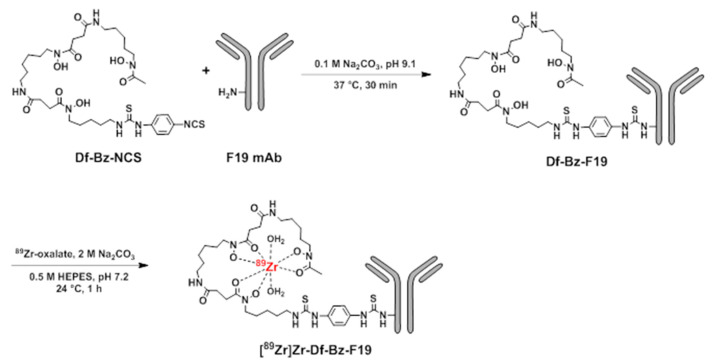
Preparation of [^89^Zr]Zr-Df-Bz-F19 mAb.

**Figure 1:**
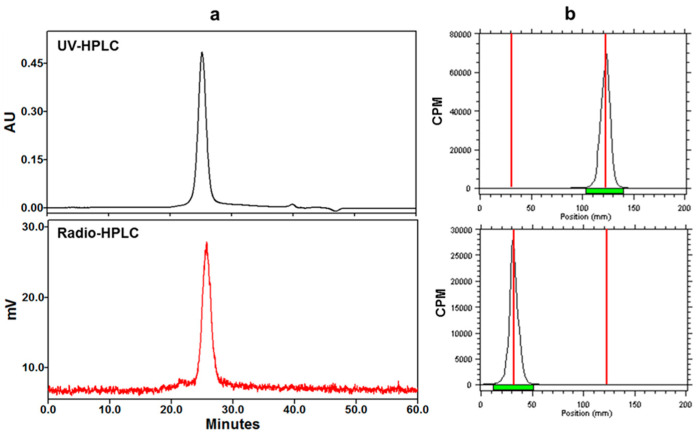
**Quality control of [^89^Zr]Zr-Df-Bz-F19.** (**a**) UV-HPLC chromatogram (280 nm) of Df-Bz-F19 (top) compared with radio-HPLC chromatogram of [^89^Zr]Zr-Df-Bz-F19 (bottom). Chromatograms demonstrate that [^89^Zr]Zr-Df-Bz-F19 can be prepared with high purity. The radiotracer also demonstrates the same retention time as the unlabeled antibody; (**b**) Radio-ITLC of [^89^Zr]Zr(ox)_2_ (top), and [^89^Zr]Zr-Df-Bz-F19 (bottom). In this ITLC-SG system, unchelated [^89^Zr]Zr(ox)_2_ was complexed by the eluent EDTA to form [^89^Zr]Zr-EDTA, which eluted with the solvent front (R_f_ ~ 1), while [^89^Zr]Zr-Df-Bz-F19 remained at the origin (R_f_ ~ 0).

DF-Bz-F19 was prepared using standard isothiocyanate chemistry, which involved the reaction of the NCS group of Df-Bz-NCS with available NH_2_ groups of F19 mAb (Scheme 1). Immunoconjugation was achieved by adding a 5-fold molar excess of Df-Bz-NCS, and purified by PD-10 column with saline (0.9% NaCl). A single peak at 25.2 min in the UV chromatogram demonstrated that the conjugate had high purity (Figure 1a top).

The Df-Bz-F19 conjugate was radiolabeled quantitatively by incubation with ^89^Zr(ox)_2_ in 0.5 M HEPES buffer (pH 7.2) at room temperature for 1 h with a radiochemical purity of ≥99.5% (n = 15). Based on radio-HPLC analysis (Figure 1a bottom), the radiopharmaceutical had a retention time of 25.7 min., like the retention time of Df-Bz-F19, suggesting formation of the radiopharmaceutical. In the radio-TLC analysis, ^89^Zr, which was not incorporated into the immunoconjugate, formed a complex with EDTA and eluted with solvent front (Figure 1b top) while [^89^Zr]Zr-Df-Bz-F19 conjugate remained at the origin (Figure 1b bottom). The specific activity of [^89^Zr]Zr-Df-Bz-F19 was calculated as 158.3 ± 1.6 MBq/mg (4.28 ± 0.04 mCi/mg; n = 4). [^89^Zr]Zr-Df-Bz-F19 showed less than 4% of ^89^Zr transchelation in human serum after 7 days at physiological temperature (Supplementary Figure S1), suggesting it would be stable upon in vivo injection. Using the FAP^+^ U87MG cell line and Lindmo analysis, [^89^Zr]Zr-Df-Bz-F19 had a K_a_ of 3.03 × 10^−8^ M^−1^ (2.76 × 10^−8^-3.42 × 10^−8^ M^−1^ 95% confidence interval [CI]), a B_max_ of 1.5 × 10^5^ fmol/mg (1.5 × 10^5^–1.68 × 10^5^ fmol/mg 95% CI), and an immunoreactivity (IR) of 52%.

### 2.2. Biodistribution Studies

**Figure 2:**
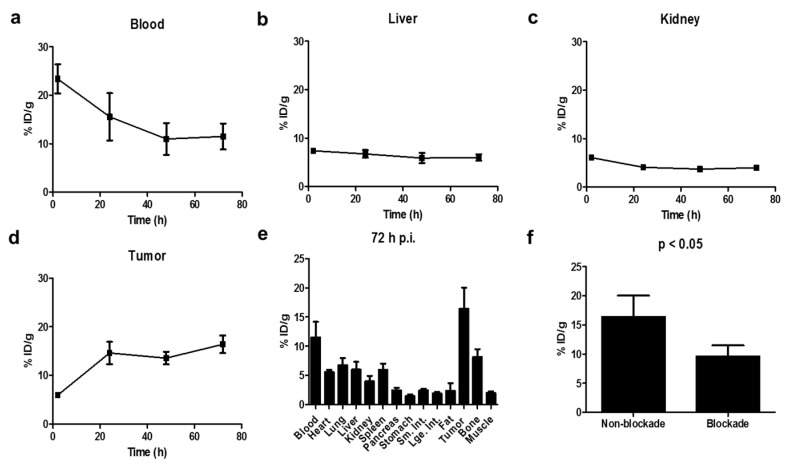
Biodistribution data of [^89^Zr]Zr-Df-Bz-F19 in select tissues: (**a**) blood; (**b**) liver; (**c**) kidney; (**d**) U87MG tumor; (**e**) The complete biodistribution profile of [^89^Zr]Zr-Df-Bz-F19 at 72 h p.i.; (**f**) The influence of F19 mAb blockade on the retention of [^89^Zr]Zr-Df-Bz-F19 in tumor tissue.

[^89^Zr]Zr-Df-Bz-F19 showed modest blood clearance, with 51% of the activity present at 2 h removed from the blood by 72 h (Figure 2 and Supplementary Table S1). In contrast, slower clearance occurred in the liver and kidney. From 2–72 h, activity in the liver decreased by 19% (*p* = 0.17), while 35% (*p* = 0.01) of the 2 h activity was excreted from the kidney by the end of the study. Interestingly, a 30% increase in ^89^Zr accumulation in bone was observed by 72 h compared to the 2 h time point.

Initial accumulation in the tumor was modest at 2 h, but increased by 175% after 72 h. This yielded average tumor-to-blood ratios of 0.25, 0.93, 1.2, and 1.4 at 2, 24, 48, and 72 h, respectively, and average tumor-to-muscle ratios of 3.1, 7.3, 7.2, and 8.3 at 2, 24, 48, and 72 h, respectively. Measurements in blocking studies were performed at 72 h post-injection by co-injecting the non-radioactive F19 mAb 2 h before injecting [^89^Zr]Zr-Df-Bz-F19 (Figure 1f). F19 blockade reduced the accumulation of [^89^Zr]Zr-Df-Bz-F19 in the tumor by 41% at 72 h (*p* < 0.05). At 72 h, blockade reduced the tumor-to-blood and tumor-to-muscle ratios to 0.84 and 5.6, respectively.

### 2.3. Small Animal Imaging Studies

**Figure 3:**
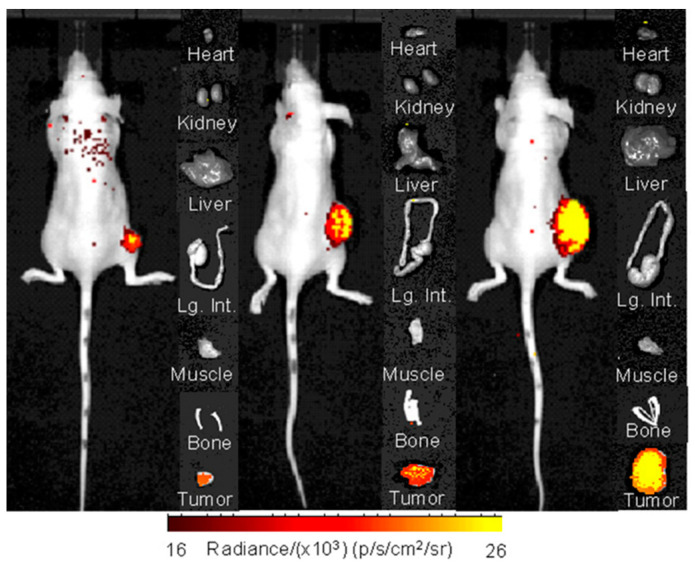
Cerenkov luminescence imaging of FAP^+^ U87MG tumor-bearing mice at 72 h p.i. using [^89^Zr]Zr-Df-Bz-F19.

Cerenkov luminescence imaging (CLI) experiments using [^89^Zr]Zr-Df-Bz-F19 indicated increasing luminescence intensity among the FAP^+^ tumors; background signal gradually decreased over time due to systemic clearance of [^89^Zr]Zr-Df-Bz-F19 (Figure 3). Ex vivo organ imaging confirmed the in vivo results. Based on ROI analysis, FAP^+^ tumors had an average radiance of 8.5 × 10^3^ ± 1.5 × 10^3^ p/s/cm^2^/sr. FAP^−^ tissues such as muscle displayed an average radiance not exceeding background levels.

**Figure 4:**
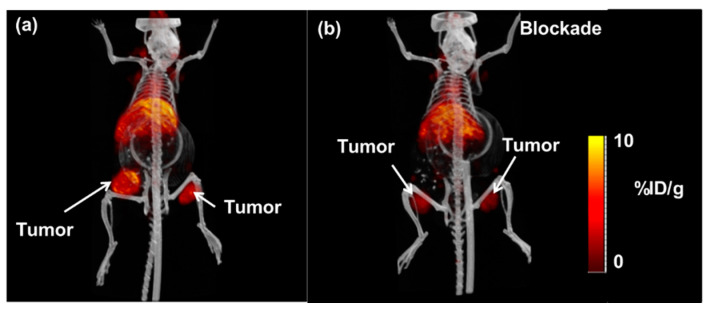
(**a**) Whole body volume rendered PET/CT image (72 h p.i.) of an animal receiving [^89^Zr]Zr-Df-Bz-F19, showing tumor retention of the radiotracer; (**b**) whole body volume rendered PET/CT image (72 h p.i.) of an animal receiving [^89^Zr]Zr-Df-Bz-F19 and F19 mAb blockade. Radioactivity within tumors is significantly reduced upon blockade. *Arrows* indicate position of tumor.

**Figure 5:**
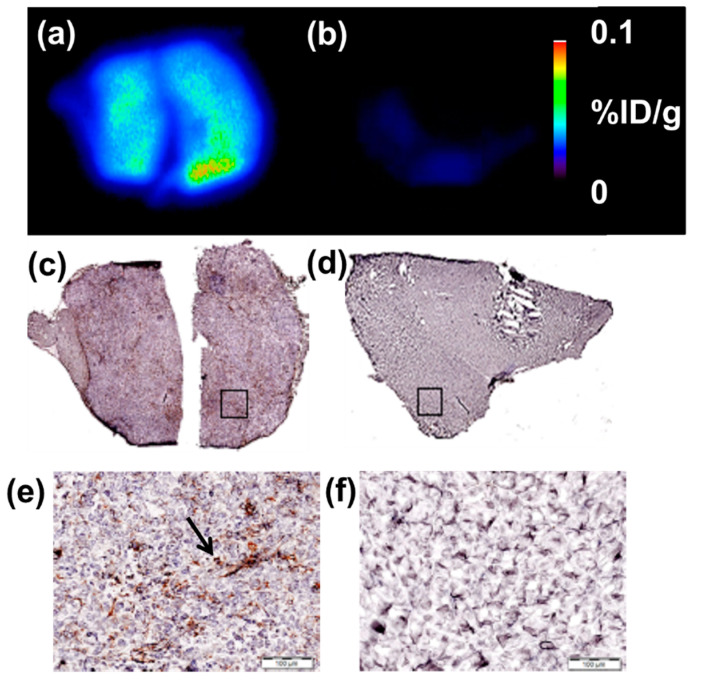
Autoradiography and histology confirm the presence of [^89^Zr]Zr-Df-Bz-F19 in only FAP^+^ tissues. (**a**) Autoradiography corroborates results of small animal imaging experiments; radioactivity is localized in the FAP^+^ tumor; (**b**) FAP^−^ tissues (e.g., muscle) demonstrated non-specific and minimal accumulation of radioactivity; Macroscopic view of sequential tumor (**c**) and muscle (**d**) slices used to corroborate autoradiographic studies; black squares indicate spatial area where tissue magnification (100×) occurred to observe the presence (**e**) or absence (**f**) of FAP expression in each tissue. Arrows indicate FAP expression.

Figure 4a displays a representative result of the volume rendered PET/CT image of an animal receiving [^89^Zr]Zr-Df-Bz-F19. Localization and increasing accumulation of radioactivity was seen within FAP^+^ tumors when compared to FAP^−^ tissues (e.g., muscle) during the same time course (Supplementary Figure S2). However, radioactivity within tumors was significantly decreased upon the administration of F19 mAb blockade (Figure 4b). Without blockade, tumor-to-muscle ratios at 2, 24, 48, and 72 h were 2.0, 5.0, 6.1, and 7.8, respectively. Similar to the biodistribution results, activity was still observed in the blood pool and within the hepatobiliary system 72 h after the injection of [^89^Zr]Zr-Df-Bz-F19. Representative results of autoradiography (AR) and immunohistochemistry (IHC) analyses are shown in Figure 5a–f. AR demonstrated the accumulation and retention of activity throughout the interior of the tumor. IHC staining for FAP antigen in sequential tumor sections confirmed the presence of FAP within regions of the tumor, which also demonstrated increased radioactivity accumulation. In contrast, FAP^−^ muscle showed non-specific retention of radioactivity barely above background levels, and IHC demonstrated no FAP expression in FAP^−^ tissues (e.g., muscle) corroborating the autoradiography results.

## 3. Discussion

The restricted expression profile of FAP in normal adult tissues along with its overexpression in a variety of pathologies has intensified research efforts that involve the development of anti-FAP therapies and new imaging agents to detect and quantify FAP expression in vivo. As our initial foray in this area, we prepared the PET radiopharmaceutical, [^89^Zr]Zr-Df-Bz-F19 with high radiochemical purity and a specific activity comparable to other ^89^Zr-Df immunoconjugates [45,46,47]. Lindmo analysis revealed that [^89^Zr]Zr-Df-Bz-F19 displayed an affinity for the FAP protease, in agreement with other anti-FAP antibody constructs previously reported [6,16,17,19,20], but demonstrated a modest immunoreactivity, probably caused by altered antigen binding due to the non-selective conjugation of Df-Bz-NCS to the antibody. However, we did not attempt to control thiourea bond formation during Df-Bz-NCS conjugation, since the radiopharmaceutical’s affinity for FAP and the large B_max_ observed with the U87MG cell line was considered sufficient for effective tumor targeting [48].

Clearance and retention properties of [^89^Zr]Zr-Df-Bz-F19 were investigated through biodistribution studies using a xenotransplantation model consisting of nude mice bearing FAP^+^ U87MG tumors. Although numerous models have used tumors derived from cells engineered to express FAP [49], we chose the U87MG cell line since it endogenously expresses FAP and has been used successfully in previous reports to evaluate probes, which target FAP in vivo [11,12,37]. Furthermore, a cell line with endogenous FAP expression provides a more realistic evaluation of our radiopharmaceutical after injection.

[^89^Zr]Zr-Df-Bz-F19 effectively targeted cell surface FAP expression with specificity since retained radioactivity was reduced in tumors of animals receiving blockade. Radioactivity retention within the tumor increased rapidly between 2 and 24 h, but only slightly thereafter, suggesting that saturation of antigenic binding sites occurs early in the experimental time course.

Tissue distribution profiles consistent with radiolabeled mAbs included slow clearance of the radiotracer from the blood pool and retention of activity throughout the gut, suggesting hepatobiliary clearance [17,19,20,45,46,47]. Surprisingly, significant amounts of radioactivity were retained in the kidney and in the bone after 72 h and suggests that [^89^Zr]Zr-Df-Bz-F19 is being metabolized over time in vivo. This behavior was observed with other ^89^Zr-labeled immunoconjugates and may be attributed to murine metabolism or the less selective nature of murine proteases [40,41]. ^89^Zr^4+^ ions sequestered by phosphate-rich hydroxyapatite probably results in the appreciable activity retained in bone, while kidney retention may result from catabolism of the radiopharmaceutical after interaction with the Fc receptor [50]. Once catabolism occurs, the decomplexed ^89^Zr^4+^ion may electrostatically interact with the glomerular basement membrane, which is composed of polyanionic heparin sulfate [51,52]. Additionally, given the abundance of phosphate ions within the kidney, ^89^Zr (IV) phosphates may also be precipitating in this tissue and leading to radioactivity accumulation there. However, other chemical species and retention mechanisms, either alone or in concert, cannot be ruled out in either site. Regardless, these results suggest radiometal chelate instability and reinforce the idea that new bifunctional chelators—which can chelate ^89^Zr under mild conditions, form kinetically and thermodynamically inert complexes, and withstand the proteolytic environment in vivo—are needed to reduce accumulation of this radiometal in non-target tissues [40,53].

Cerenkov luminescence imaging (CLI), which synergizes nuclear medicine and optical imaging continues to evolve as associated technologies mature and are integrated into preclinical and clinical applications [42,43,44,54,55]. Preclinically, it can augment the information that is provided through biodistribution and small animal PET/CT studies and aids in biomarker discovery and the drug development process. Accordingly, we exploited the prompt release of Cerenkov radiation attributed to positron emission from the ^89^Zr nucleus to image FAP expression using CLI [44,53]. CLI discriminated between FAP^+^ (tumor) and FAP^−^ (muscle) tissues and FAP^+^ tumors of varying sizes. Consistent with other studies, tumor visualization became more efficient over time due to the clearance of the circulating radiopharmaceutical from the blood pool and non-target tissues, which was responsible for elevated background during early time points of this study [45,46,47]. Ex vivo imaging of the FAP^+^ tumor and organs was also performed to investigate the effects that attenuation and scattering of Cerenkov radiation may have had on the observed imaging results obtained with whole animals. Additionally, gamma counting of excised tissues revealed that FAP^+^ tumors retained the greatest amount of radioactivity. These ex vivo results correlated well with the in vivo results; most radioactivity was retained in the FAP^+^ tumors.

We also conducted the small animal PET/CT imaging of animals bearing FAP^+^ tumors to more accurately quantify radiopharmaceutical localization in FAP^+^ and FAP^−^ tissues. Tumor retention and accumulation of radioactivity was evident at every time point in animals receiving [^89^Zr]Zr-Df-Bz-F19. In accordance with our biodistribution results, [^89^Zr]Zr-Df-Bz-F19 accumulated rapidly during the first 24 h of the study (Supplementary Figure S2). Increasingly efficient contrast, which was demonstrated by increasing tumor-to-muscle ratios, supported efficient tumor targeting and clearance of [^89^Zr]Zr-Df-Bz-F19 over time. The specificity of our radiopharmaceutical for FAP was also confirmed by autoradiography and immunohistochemistry performed on FAP^+^ and FAP^−^ tissues excised at the completion of the in vivo imaging studies. These results demonstrate that localization of [^89^Zr]Zr-Df-Bz-F19 was mainly confined to FAP^+^ tissues, while FAP^−^ tissues retained very little radioactivity. Recent publications have described a more ubiquitous expression of FAP in murine models of cancer, and since murine and human FAP share 89% sequence homology including the catalytic active site, we expected to observe reduced tumor-to-non-target tissue contrast in our in vivo imaging studies [56]. However, this was not the case as FAP^+^ tumors are clearly defined and distinct from the surrounding flank muscles, which demonstrate negligible FAP expression. These results further corroborate literature describing limited in vivo expression of this biomarker in normal tissues [4,5].

Several limitations to these studies should be considered despite the positive results. Although other protein-based, anti-FAP agents exist [6,17,19,20], our initial investigations into FAP imaging involved the use of the F19 mAb. Although it failed in clinical trials more than two decades ago and has since been marginalized as an anti-FAP mAb by other novel agents [6,15], we chose this mAb because it is commercially available, could be produced in large quantities using hybridoma technology and allowed us to probe FAP expression on glioma cells in a facile manner. Secondly, the use of a mAb-based agent for imaging neuroepithelial tumors may also seem to be a limitation due to the protective nature of the blood—brain barrier (BBB). While the BBB, a natural defensive mechanism, may regulate central nervous system homeostasis and maintain normal brain function, it may also impede the delivery of imaging agents and systemic therapies to brain tumors [57]. However, upon neuroepithelial tumor cell invasion, the BBB is often compromised due to the invasive and aggressive nature of these tumors. Once compromised, large molecules such as mAbs may be able to traverse this barrier although admittedly, in an inefficient manner [3,58]. Furthermore, previous research has demonstrated that the BBB can be permeabilized using several techniques including high-intensity focused ultrasound, external beam radiation and systemic radiotherapy to enhance the administration of agents across this barrier to improve diagnosis and therapy [59,60,61,62,63,64]. Furthermore, these investigations did not use an orthotopic tumor model of GBM. While such a model may recapitulate various aspects of disease more accurately than the subcutaneous tumor model used here, we chose the latter since it allowed us to study FAP targeting without the experimental complexity associated with intracranial tumor implantation. Attempts to extrapolate our findings in an orthotopic paradigm are currently underway in our laboratory. Finally, while CLI did visualize luminescence in FAP^+^ tissues and not in FAP^−^ tissues, gamma counting did detect radioactivity in all excised tissues. This result illustrates the technical hurdles relating to detection sensitivity that can be associated with this imaging modality. Despite this limitation, advances in CLI technology and techniques continue to be made not only for diagnostic imaging but also for image-guided drug delivery and intra-operative guidance [42,43,44,65,66,67,68,69,70]. Currently multi-modal systems exist to detect malignancy though optical and radiometric detection [71,72,73,74]. As technologies and techniques are refined in this research area, it is entirely plausible that radiometric and CLI-enhanced detection have a role in providing intra-operative guidance within the context of neuroepithelia tumor resection.

## 4. Materials and Methods

### 4.1. Reagents and Equipment

Zirconium-89 (^89^Zr: t_½_ = 78.4 h, β^+^: 22.8%) was purchased from Washington University School of Medicine (St. Louis, MO, USA) or Sophie Biosciences, Inc. (Dulles, VA, USA) [75]. Unless noted, all other chemicals were purchased from Sigma-Aldrich Chemical Co. (St. Louis, MO, USA), and solutions were prepared using ultrapure water (18 MΩ-cm resistivity). p-isothiocyanatobenzyl-desferrioxamine (Df-Bz-NCS) was purchased from Macrocyclics, Inc. (Dallas, TX, USA). The anti-FAP F19 antibody was obtained through hybridoma technology (ATCC, Manassas, VA, USA) and purified using standard techniques [76,77]. Radiochemistry reaction progress and purity were monitored using analytical high-performance liquid chromatography (HPLC) (Waters, Milford, MA, USA), which runs Empower^3^ software and is configured with a 1525 binary pump, 2707 autosampler, 2998 photodiode array detector, 2475 multichannel fluorescence detector, 1500 column heater, fraction collector, size exclusion Superdex 200 10/300 GL (GE Healthcare Life Sciences, Piscataway, NJ, USA) column, a Carrol Ramsey 105-s radioactivity detector (Berkeley, CA, USA), and an isocratic mobile phase (0.5 mL/min) consisting of phosphate buffered saline (PBS, pH 7.1 (NaCl 150 mM, Na_2_HPO_4_ 50 mM, NaH_2_PO_4_ 50 mM, and NaN_3_ 10 mM)). Radio-TLC analysis was performed on Bioscan AR 2000 radio-TLC scanner equipped with a 10% methane: argon gas supply, a PC interface running Winscan v.3 analysis software (Eckert & Ziegler, Berlin, Germany), and Varian ITLC-SG strips (Agilent Technologies, Santa Clara, CA, USA), with 50 mM EDTA (pH 5) as eluent. Radioactive samples were counted using a Perkin Elmer 2480 Wizard^®^ gamma counter (Waltham, MA, USA) with an energy window of 500–1500 keV. PET and CT images were acquired using a GE eXplore Vista small animal PET/CT scanner (Waukesha, WI, USA).

### 4.2. Conjugation, ^89^Zr-Radiolabeling, and Quality Control

[^89^Zr]Zr-Df-Bz-F19 was prepared using a modified procedure [45,47]. Briefly, F19 mAb (4 mg in 500 µL PBS, pH 7.4) was diluted to 1 mL with normal saline, and the pH adjusted to pH 8.9–9.1 with 0.1 M Na_2_CO_3_ (90 µL). Df-Bz-NCS (8 mM; 15 μL of DMSO) was added and the resulting solution was incubated for 30 min at 37 °C using a thermomixer at 550 r.p.m. To remove non-conjugated Df-Bz-NCS, Df-Bz-F19 was purified by PD-10 column using saline (0.9% NaCl). The purified Df-Bz-F19 conjugate was stored at 4 °C and used for ^89^Zr-radiochemistry.

[^89^Zr]Zr-oxalate (74−93 MBq in 100–125 µL 1.0 M oxalic acid) and 2 M Na_2_CO_3_ (40–50 µL), were placed in a 1.5 mL tube and incubated at room temperature for 3 min followed by pH adjustment to 6.8–7.2 using 0.5 M HEPES buffer (400 μL, pH 7.2). Gentisic acid (100 µL, 5 mg/mL in 0.25 M NaOAc) and 0.3–0.4 mg of Df-Bz-F19 conjugate in PBS buffer (75–100 µL) were then added; the resulting mixture was incubated at room temperature for 1 h. The reaction was quenched with ethylenediaminetetraacetic acid solution (10 μL, 50 mM EDTA), and [^89^Zr]Zr-DF-Bz-F19 was purified using PD-10 column with 0.25 M sodium acetate/gentisic acid (5 mg/mL) buffer (pH 5.5) as eluent. Radiochemical yield and purity were determined by radio-TLC and HPLC.

### 4.3. In Vitro Serum Stability of [^89^Zr]Zr-Df-Bz-F19

In vitro stability was carried out by adding 10 µL of [^89^Zr]Zr-Df-Bz-F19 (50 μCi, 1.85 MBq) to 500 µL human serum. The solutions (n = 3) were incubated at 37 °C for 7 days and analyzed daily by radio-TLC using a mobile phase consisting of 50 mM EDTA (pH 5) on Varian ITLC-SG strips [45,47].

### 4.4. Immunoreactivity (IR) Studies

Immunoreactivity of [^89^Zr]Zr-Df-Bz-F19 to FAP^+^ U87MG cells was determined by the Lindmo method [78]. Briefly, 50 ng of [^89^Zr]Zr -Df-Bz-F19 were added to a range of cell concentrations and incubated for 60 min at 4 °C with continuous mixing. Cells were washed three times, pelleted using centrifugation, and the activity within the pellet was measured by gamma counting. Three samples of [^89^Zr]Zr-Df-Bz-F19 (at the same concentration as that initially added to the cells) were measured at the same time as cell pellets. A background correction was applied by adding 100-fold unlabeled F19 antibody to three tubes containing cells before addition of [^89^Zr]Zr-Df-Bz-F19. The percentage of [^89^Zr]Zr-Df-Bz-F19 binding to U87MG cells was calculated ((cpm cell pellet/mean cpm [^89^Zr]Zr-Df-Bz-F19 standards) × 100), and the percent binding was plotted as a function of cell concentration using GraphPad Prism 5.0 software (San Diego, CA, USA). Immunoreactivity (IR) was calculated from the Y-intercept of the inverse plot of both values.

The association constant (K_a_) and the number of antibody molecules bound per cell (B_max_) were determined using Scatchard analysis. Varying concentrations (0.01–8 µM) were added to 2 × 10^6^ U87MG cells and mixed before adding 50 ng of [^89^Zr]Zr-Df-Bz-F19. After 1 h incubation at 4 °C with continuous mixing, the cells were washed three times and counted as described above. The free, reactive antibody was calculated using the formula: [(100 − %bound)/100 × total antibody × IR fraction]. Specific binding (nM): [total antibody × %bound] was graphed against specific binding/reactive free antibody. The association constant was determined from the negative slope of the line. The number of [^89^Zr]Zr-Df-Bz-F19 molecules bound per cell (B_max_) was derived from the formula: [X-intercept of Scatchard plot (nM)/1000 × 6.02 × 10^23^)/2 × 10^6^ cells].

### 4.5. Xenograft Models

Animal Work was approved by the Wake Forest University Health Sciences and University of Iowa Institutional Animal Care and Use Committees under protocols A17-062 (approval dates 5 May 2017–4 May 2020) and 0012266 (approval dates 8 May 2018–7 May 2021), respectively. Female athymic nu/nu mice (6–8 wks) were obtained from Jackson Laboratories (Bar Harbor, ME, USA). U87MG tumor cells (ATCC) were cultured in ATCC-formulated Eagle’s Minimum Essential Medium (ATCC No. 30-2003). Once at 80% confluency, 1 × 10^6^ cells in 100 µL serum-free culture medium were mixed with Matrigel^®^ (BD Biosciences, San Jose, CA, USA) and subcutaneously injected into the flank. Cell growth was evaluated weekly using manual tumor volume (volume = 0.52 × [width]^2^ × [length]) measurements.

### 4.6. Biodistribution

Biodistribution studies were conducted as previously described [45,47,79]. Tumor-bearing mice (n = 6/group) were injected with [^89^Zr]Zr-Df-Bz-F19 (0.69–0.74 MBq, 3–3.5 µg in 150 µL PBS/mouse) via the tail vein. Blocking studies were also performed whereby animals in this cohort received F19 mAb (0.20 mg/mouse), 2 h before being injected with [^89^Zr]Zr-Df-Bz-F19. Animals were then sacrificed at 2, 24, 48 and 72 h post-injection (p.i.). Blood, heart, lungs, liver, kidney, spleen, pancreas, stomach, large intestine, small intestine, muscle, fat, bone and tumor were removed, weighed, and subjected to gamma counting. The percent injected dose per gram (%ID/gram) and percent injected dose per organ (%ID/organ) were calculated by comparison to a weighed, counted standard.

### 4.7. Small Animal Optical Imaging Using Cerenkov Luminescence

Imaging studies were conducted using a modified method [54,67]. Briefly, all tumor-bearing mice received an injection of [^89^Zr]Zr-Df-Bz-F19 (10.2–10.5 MBq, 48–50 µg in 150 µL PBS/mouse) via the tail vein. Mice were anesthetized with 1–2% isoflurane and imaged at 2, 24, 48 and 72 h p.i. After the 72 h time point, animals were euthanized, and tumors and organs of interest were removed and imaged ex vivo. Optical images were collected using a Xenogen IVIS 100 optical imager (f/stop: 2; binning 1, filed of view B) with no light interference from the excitation lamp. Images were analyzed using Living Image 2.6 software (Caliper Life Sciences, Alameda, CA, USA). The average radiance (p/s/cm^2^/sr) was used for quantitative region of interest (ROI) analysis from each image. Background correction was performed either through use of dark images acquired at the equivalent instrument integration setting immediately before experimental image collection, or by subtracting background levels in the same experimental image but remote from the area of interest.

### 4.8. Small Animal PET/CT Imaging

All tumor-bearing mice (n = 6/group) received an injection of [^89^Zr]Zr-Df-Bz-F19 (7.9–10.2 MBq, 48–50 µg in 150 µL saline/mouse) via the tail vein. Mice were anesthetized with 1–2% isoflurane and imaged for 30 min at 2, 24, 48, and 72 h p.i. Images were reconstructed using ordered subset expectation maximum (OSEM) algorithms, coregistered with the CT image, and the percent injected dose of activity per gram of tissue (%ID/g) was determined at every time point using known procedures [45,47].

### 4.9. Autoradiography (AR) and Immunohistochemistry (IHC)

Animals were sacrificed and FAP^+^ tumors and FAP^−^ muscle were excised, frozen, embedded in OCT medium and sectioned on a CM1850 cryo-microtome (Leica Microsystems, Inc. Chicago, IL, USA). Tissue sections were fixed in ice-cold acetone for 60 s, allowed to dry and then placed in contact with a BAS-IP MS 2040 E multipurpose phosphor screen inside a standard cassette for 72 h at −80 °C. The screen was imaged using a Typhoon 9210 Variable Mode Imager (Molecular Devices, Sunnyvale, CA, USA) according to an established protocol [80]. Sequential 8 μm sections were used for IHC analysis. Tumor and muscle sections were exposed to rabbit-derived, anti-human FAP mAb (Abcam, Cambridge, UK) and developed using 3,3′-diaminobenzidine (DAB) according to an established protocol [81]. An isotype-matched human IgG control antibody and no primary antibody controls were used for each tissue.

### 4.10. Statistical Methods

All data are presented as mean ± SD or mean (95% confidence intervals). For statistical classification, a Student’s t test (two-tailed, unpaired) was performed using GraphPad Prism software. Any difference of *p* < 0.05 was considered significant.

## 5. Conclusions

The PET radiopharmaceutical [^89^Zr]Zr-Df-Bz-F19 was evaluated in vitro and in vivo with the U87MG cell line, which endogenously expresses FAP. Despite moderate immunoreactivity, efficient tumor uptake and excellent tumor-to-background contrast was achieved in CLI and small animal PET/CT studies. FAP represents a promising biomarker that can be exploited to target a variety of pathological conditions and possibly the tumor microenvironment using PET.

## Supplementary Materials

The following are available online, Figure S1. In vitro serum stability of [^89^Zr]Zr-Df-Bz-F19. Figure S2. Standard uptake value quantification of [^89^Zr]Zr-Df-Bz-F19mAb from PET/CT imaging study. Figure S3. Binding Data. Table S1. Biodistribution of [^89^Zr]Zr-Df-Bz-F19 in U87MG tumor bearing mice.
