# Supplementary material for: Imaging of Fibroblast Activation Protein Alpha Expression in a Preclinical Mouse Model of Glioma Using Positron Emission Tomography
Source: Molecules. 2020 Aug 12;25(16):3672. doi: 10.3390/molecules25163672 (PMC7464128; doi:10.3390/molecules25163672)
Supplement: Supplementary file 1 [file molecules-25-03672-s001.pdf]

Supplementary Information

Imaging of Fibroblast Activation Protein Alpha Expression in a Preclinical Mouse Model of Glioblastoma Using Positron Emission Tomography

Darpan N. Pandya[1], Akesh Sinha[1], Hong Yuan[2], Lysette Mutkus[3], Kristina Stumpf[3], Frank C. Marini[3] and Thaddeus J. Wadas*[1]

[1]Department of Radiology, University of Iowa, Iowa City, IA 52242 USA; darpan-pandya@uiowa.edu (D.N.P.); akesh-sinha@uiowa.edu (A.S.); thaddeus-wadas@uiowa.edu (T.W.)

[2]Department of Radiology, University of North Carolina at Chapel Hill, Chapel Hill, NC 27599 USA; yuanh@med.unc.edu (H.Y.)

[3]Department of Regenerative Medicine, Wake Forest University Health Sciences, Winston-Salem, NC 27157 USA; lmutkus@wakehealth.edu (L.M.); kstumpf@wakehealth.edu (K.S.); fmarini@wakehealth.edu (F.M.)

* Correspondence: thaddeus-wadas@uiowa.edu; Tel.: +01-319-335-5009

Table of Contents

Section	Page Number
**Figure S1.** ***In vitro*** **serum stability of [^{89}Zr]Zr-Df-Bz-F19**	3
**Table S1. Biodistribution of [^{89}Zr]Zr-Df-Bz-F19 in U87MG tumor bearing mice**	4
**Figure S2. Standard uptake value quantification of [^{89}Zr]Zr-Df-Bz-F19 from PET/CT imaging study**	5
**Figure S3. Binding data of [^{89}Zr]Zr-Df-Bz-F19 mAb**	6

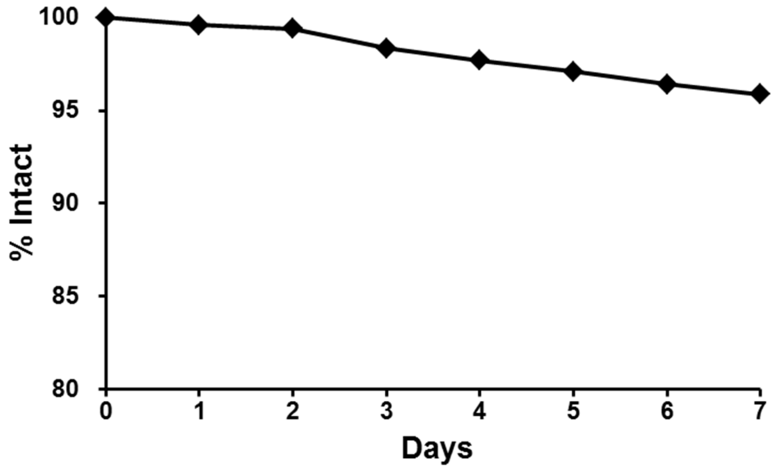

Figure S1. ***In vitro*** **serum stability of [^{89}Zr]Zr-Df-Bz-F19.** Serum stability studies (n = 3) demonstrate that the radiopharmaceutical remains more that 96% intact after 7 days in human serum at physiological temperature.

Table S1. Biodistribution of [^{89}Zr]Zr-Df-Bz-F19 in U87MG tumor bearing mice (n ≥ 4; %/ID/g)

Tissue/Organ	2 h	24 h	48 h	72 h	72 h Blockade
Blood	23.72 ± 2.92	15.54 ± 5.43	10.74 ± 3.70	11.11 ± 3.10	11.31 ± 2.70
Heart	11.25 ± 0.91	7.40 ± 1.32	5.09 ± 1.42	5.58 ± 1.04	5.89 ± 1.75
Lung	16.38 ± 2.85	8.17 ± 2.20	6.35 ± 1.33	6.96 ± 1.79	7.11 ± 0.91
Liver	7.12 ± 1.40	6.81 ± 1.48	6.13 ± 2.48	6.06 ± 1.78	5.89 ± 0.88
Kidney	5.82 ± 0.85	4.11 ± 0.74	3.56 ± 0.67	4.05 ± 1.26	3.85 ± 0.41
Spleen	6.64 ± 0.50	5.72 ± 1.75	5.49 ± 1.62	6.01 ± 1.45	5.09 ± 1.54
Muscle	1.92 ± 0.35	1.98 ± 0.26	1.87 ± 0.30	1.97 ± 0.44	1.74 ± 0.34
Bone	5.72 ± 1.62	6.53 ± 1.32	4.79 ± 1.79	8.33 ± 1.45	7.24 ± 1.67
Tumor	5.94 ± 0.84	14.61 ± 3.99	13.56 ± 2.62	16.40 ± 3.65	9.60 ± 1.90
Pancreas	3.20 ± 0.41	3.28 ± 1.20	2.30 ± 0.61	2.59 ± 0.81	2.24 ± 0.61
Stomach	1.36 ± 0.50	1.84 ± .040	1.10 ± 0.30	1.53 ± 0.53	1.53 ± 0.37
Large Intestine	3.49 ± 0.32	3.24 ± 1.07	2.10 ± 0.47	1.90 ± 0.44	2.00 ± 0.29
Small Intestine	3.86 ± 0.84	3.44 ± 0.59	2.37 ± 0.58	2.65 ± 0.89	2.31 ± 0.32

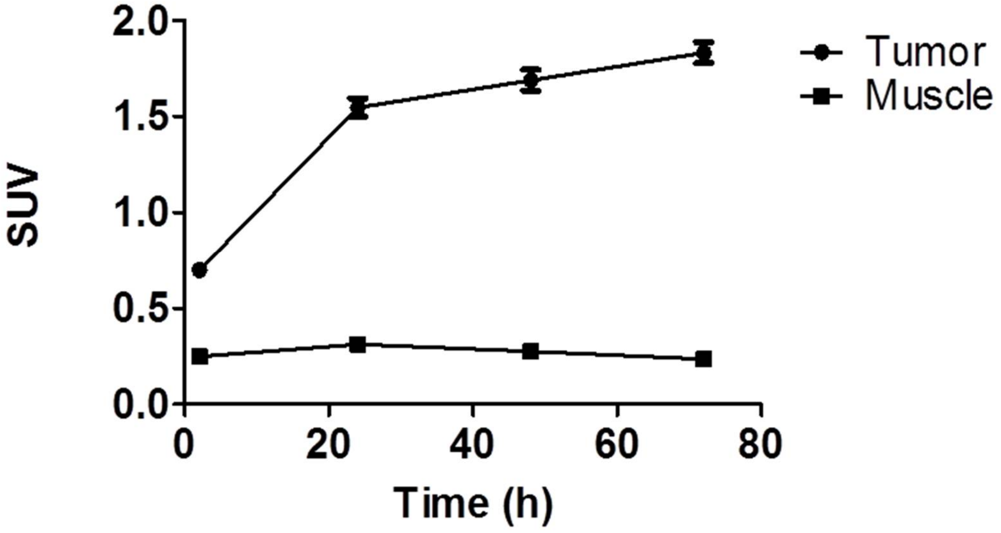

Figure S2. Standard uptake value quantification of [^{89}Zr]Zr-Df-Bz-F19 from PET/CT imaging study. [^{89}Zr]Zr-Df-Bz-F19 was retained in FAP^{+} tumor tissue but not FAP^{-} tissues such as muscle over the 72 h time course.

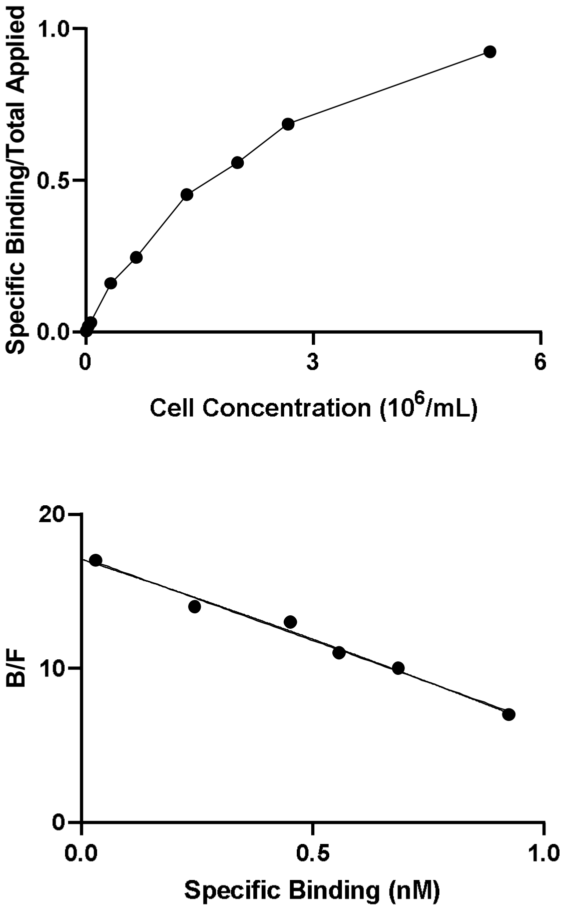

Figure S3. Binding data of [^{89}Zr]Zr-Df-Bz-F19 mAb.
